# Dietary supplementation of compound probiotics to improve performance, egg quality, biochemical parameters and intestinal morphology of laying hens

**DOI:** 10.3389/fvets.2024.1505151

**Published:** 2024-12-24

**Authors:** Yan Wang, Chaosheng Zhang, Xing Chen, Aijuan Zheng, Guohua Liu, Ying Ren, Zhimin Chen

**Affiliations:** ^1^Hubei Key Laboratory of Animal Nutrition and Feed Science, Wuhan Polytechnic University, Wuhan, China; ^2^Key Laboratory for Feed Biotechnology of the Ministry of Agriculture and Rural Affairs, Institute of Feed Research, Chinese Academy of Agriculture Sciences, Beijing, China

**Keywords:** compound probiotics, laying hens, egg quality, biochemical parameters, intestinal morphology and technology

## Abstract

The purpose of this study was to investigate the effects of dietary supplementation of compound probiotics on the performance, egg quality, biochemical parameters and intestinal morphology of laying hens. A total of 180 healthy 200-day-old Hyline Brown laying hens with similar initial laying rate (87.5% ± 0.2%) were randomly divided into the control group and the treatment group. Each group included 6 replicates and each replicate included 15 laying hens. The control group was provided a basal diet, while the treatment group received the basal diet supplemented with compound probiotics. The experiment lasted for 52 days. The study indicated the following outcomes: (1) The laying rate (LR) and average egg weight (AEW) of laying hens in the treatment group were significantly higher than those of the control group (*p* < 0.05), whereas the feed-to-egg ratio (F/E) was significantly lower (*p* < 0.05); (2) The yolk weight (YW), egg shape index (ESI) and albumen height (AH) were significantly higher (*p* < 0.05), whereas the eggshell percentage (EP) was significantly lower (*p* < 0.05) after the dietary supplementation of compound probiotics; (3) The treatment group significantly decreased in total cholesterol (TC), triglyceride (TG), alanine aminotransferase (ALT), aspartate aminotransferase (AST), malondialdehyde (MDA), immunoglobulin A (IgA), and immunoglobulin G (IgG) levels in serum compared to the CON group (*p* < 0.05). Additionally, serum levels of total protein (TP), globulin (GLB), albumin (ALB), high-density lipoprotein (HDL), alkaline phosphatase (ALP), and total antioxidant capacity (T-AOC) were significantly higher in the treatment group (*p* < 0.05); (4) The supplementation of compound probiotics to laying hen diets led to a significant reduction in crypt depth (CD) and the ratio of villus height to crypt depth (V/C) in the jejunum compared to the CON group (*p* < 0.05). In conclusion, the supplementation of compound probiotics can regulate the body metabolism and improve the intestinal morphology, thus enhancing the antioxidant capacity and immune function of the body, which in turn improves the performance and egg quality of laying hens.

## 1 Introduction

The misuse of antibiotics has led to the widespread of resistant bacteria, posing a threat to human health, animal welfare, animal production and environmental health (1). Since July 2020, China has imposed a total ban on the addition of antibiotics to animal feeds, resulting in an urgent need to find alternatives to antibiotics to ensure the growth and health of commercial poultry. In recent years, feed additives such as plant extracts (2), probiotics (3), acidifiers (4), and antimicrobial peptides (5) have been widely used in animal husbandry in China to control pathogenic bacterial infections and improve animal growth performance.

In particular, probiotics are widely used as a potential green alternative to antibiotics due to their effective probiotics effects. As non-pathogenic organisms, probiotics are able to positively influence livestock performance and health indicators by improving blood biochemical parameters, intestinal flora, immunity, intestinal integrity and digestive enzyme activity (6, 7). The main modes of action of probiotics include the following: 1. Competition with pathogenic bacteria for attachment sites in the epithelium of the digestive tract, and when the beneficial bacteria in probiotics occupy these binding sites, a protective physical barrier is formed; 2. The ability of probiotics to produce antimicrobial substances, such as bacteriocins, to inhibit the activity of pathogenic bacteria; 3. Anaerobic bacteria are present in probiotics, and in the gut microbiota, anaerobic bacteria are the dominant flora, and these bacteria promote a low oxygen tension environment in the gut, thereby inhibiting the growth of pathogens and creating an unfavorable environment for their survival and reproduction (8–10).

The addition of probiotics to laying hen diets has been shown to improve hen performance parameters including egg production, feed factor and egg quality, disease resistance and animal welfare (11–15). It has been shown that *Bacillus subtilis* can produce a variety of extracellular enzymes such as *α*-amylase, *β*-amylase, cellulase, protease, etc., which enhances intestinal digestibility, nutrient absorption and immune function (16, 17). Macit et al. (18) reported that the addition of probiotics to feed increased the monounsaturated fatty acid content of egg yolks and improved feed factor and yolk color. Park et al. (19) demonstrated that the addition of probiotics significantly increased egg production, eggshell thickness and nutrient digestibility, and reduced ammonia emissions in laying hens. It was indicated that the addition of yeast cultures could improve egg quality and enhance feed digestibility (20). Compound probiotics consist of many different types of probiotics, and the common strains are *Bacillus*, *Lactobacillus plantarum* and *Saccharomyces cerevisiae*. By combining various probiotics in appropriate proportions, it can enhance the growth of livestock and poultry, improve antioxidant capacity and regulate immunity (21). Studies have shown that the supplementation of compound probiotics to the basal diets can improve the performance of laying hens and enhance egg quality (22). In addition, it improves the immune system by increasing the production of anti-inflammatory cytokines, decreasing intestinal permeability and inhibiting oxidative stress (23). In view of this, we hypothesized that the supplementation of compound probiotics to the diets of laying hens is effective. By evaluating the effects of compound probiotics on laying performance, egg quality, serum biochemical indexes, antioxidant capacity, immune function and intestinal morphology of laying hens, we can provide theoretical basis for the application of compound probiotics in laying hens.

## 2 Materials and methods

### 2.1 Animals, diets and experimental design

The Institute of Feed Research of the Chinese Academy of Agricultural Science’s Animal Care and Use Committee granted a license for the research based on an ethical review (Statement no. IFR-CAAS20240105, Beijing, China). A total of 180 healthy 200-day-old Hyline Brown laying hens with similar initial laying rate (87.5% ± 0.2%) were randomly divided into the control group (CON) and the treatment group (PRO). Each group included 6 replicates and each replicate included 15 laying hens. The CON received a basal diet, while the PRO received a basal diet supplemented with 0.5 g/kg of compound probiotics. And the compound probiotics were supplied into the basal diet as a powder. The compound probiotics were first mixed with the premixes and then with the other ingredients to make feed mixing uniformity higher. The basic corn-soybean meal diets were formulated to meet or exceed the nutritional requirements for laying hens calculated according to The National Research Council (NRC, 1994) recommended (Table 1). The compound probiotics were consisted of *Bacillus subtilis* (1 × 10^9^ CFU/g), *Lactobacillus plantarum* M8 (1 × 10^9^ CFU/g) and *Saccharomyces cerevisiae* (1 × 10^9^ CFU/g), an were mixed in a ratio of 1: 1:1 to form compound probiotics preparation. *Saccharomyces cerevisiae* were purchased from Angel Yeast. Co, the dosage of probiotics should follow the company’s commercial recommendations. While *Bacillus subtilis* and *Lactobacillus plantarum* M8 were screened and preserved in our laboratory, and *Lactobacillus plantarum* M8 was screened with antioxidant capacity as an index and the method of Duz et al. (24) was referred to and slightly modified, and its antioxidant capacity was shown in Table 2.

**Table 1:** Antioxidant capacity of *Lactobacillus plantarum* M8.

Items		LGG	*Lactobacillus plantarum* M8
Superoxide anion	Supernatant clearance rate%	16.91	16.81
	Suspension clearance rate%	16.70	−0.63
DPPH	Supernatant clearance rate%	63.51	71.06
	Suspension clearance rate%	8.32	4.61

LGG, Lactobacillus rhamnosus GG; DPPH, 1,1-diphenyl-2-picrylhydrazyl.

**Table 2:** Ingredients and nutrient content of experimental basal diets (%, as-is basis).

Ingredient	Contents
Corn	63.47
Soybean meal	24.49
Vegetable oil	0.26
NaCl	0.32
CaHPO_4_	1.57
Limestone	9.42
*L*-Lysine	0.03
*DL*-Methionine	0.12
Choline chloride	0.10
Premix^1^	0.23
Total	100.00
Nutrients^2^	
Metabolic Energy(ME)/(MJ/kg)	11.09
Crude Protein(CP)	15.70
Lysine(Lys)	0.76
Methionine(Met)	0.38
Met+Cystine	0.66
Ether Extract(EE)	3.01
Calcium(Ca)	4.00
Phosphorus(P)	0.57
Available Phosphorus(AP)	0.37
Moisture	12.13

1 The premix provided the following per kilogram diet: (1-21d) vitamin A 10,000 IU, vitamin D3 2,000 IU, vitamin E 10 IU, vitamin K3 2.5 mg, vitamin B1 1.8 mg, vitamin B2 4 mg, vitamin B3 50 mg, vitamin B5 11 mg, vitamin B9 0.5 mg, vitamin B12 0.7 mg, biotin 0.12 mg, Cu (as copper sulfate) 8 mg, Fe (as ferrous sulfate) 80 mg, Mn (as manganese sulfate) 60 mg, Zn (as zinc sulfate) 80 mg, Se (as sodium selenite) 0.15 mg, I (as potassium iodide) 0.35 mg. (22-42d) vitamin A 8,000 IU, vitamin D3 1,500 IU, vitamin E 8 IU, vitamin K3 2.0 mg, vitamin B1 1.5 mg, vitamin B2 3 mg, vitamin B3 40 mg, vitamin B5 9 mg, vitamin B9 0.4 mg, vitamin B12 0.6 mg, biotin 0.10 mg, Cu (as copper sulfate) 6 mg, Fe (as ferrous sulfate) 60 mg, Mn (as manganese sulfate) 50 mg, Zn (as zinc sulfate) 60 mg, Se (as sodium selenite) 0.12 mg, I (as potassium iodide) 0.30 mg.

2 Metabolic Energy, amino acids, calcium, phosphorus, and available phosphorus are calculated values, while other values are measured values.

The experiment was carried out in Dongying, Shandong Province, Lanhai Animal Husbandry Base for a total of 52d. All laying hens were raised in an environmentally controlled facilities with stereoscopic cages. Adjust test conditions such as temperature, humidity and light to meet Hyline Brown laying hens management guidelines, during the trial, they were given an unlimited supply of food and enough of clean water to drink. No medications or antibiotics were administered.

### 2.2 Performance analyses

Throughout the experiment, the amount of eggs production, egg weight and feed consumption were all precisely recorded every day, and the remaining feed weight was weighed once a week, and the laying rate (LR), average egg weight (AEW), average daily feed intake (ADFI) and feed-to-egg ratio (F/E) were calculated with the unit of repetition. The calculation formula is as follows:

$\begin{array}{l} LR\;\left(\%\right)=100\times \mathrm{total number of eggs production}\\ \;/\left(\mathrm{number of hens}\times \mathrm{experimental days}\right)\text{.} \end{array}$

$AEW\;\left(g\right)=\mathrm{total}\;\mathrm{egg}\;\mathrm{weight}/\mathrm{total number of eggs production}\text{.}$

$\begin{array}{l} \mathrm{ADFI}\;\left(g\right)=\mathrm{total feed consumption}/\\ \;\left(\mathrm{number of hens}\times \mathrm{experimental days}\right)\text{.} \end{array}$

F/E=total feed consumption/totaleggweight.

### 2.3 Egg quality

Two eggs were chosen at random from each replication after the experiment to assess the quality of the eggs. Egg analyzer (Orka Food Technology Ltd., Israel) was used to measure yolk color (YC), albumen height (AH), and Haugh unit (HU). To determine the egg shape index (ESI), the length and width of the egg are measured using an electronic digital caliper. The ESI is calculated by the formula index = egg length/ egg width. The egg force reader (Orka Food Technology Ltd., Israel) was used to test the eggshell strength (ES). The thickness of the eggshell was measured at the blunt, sharp and equator of the eggshell using the electronic digital caliper and the mean value obtained was the eggshell thickness (ET). To determine the yolk percentage (YP), albumen percentage (AP), and eggshell percentage (EP), an electronic balance was used to measure the egg weight (EW), yolk weight (YW), and egg shell weight (ESW). The formulas are as follows.

$YP\;\left(\%\right)=100\times YW/EW\text{.}$

$EP\;\left(\%\right)=100\times ESW/EW\text{.}$

### 2.4 Serum biochemistry

Two hens were removed from each replication at the conclusion of the experiment, and about 3–5 mL of blood was extracted from the wing vein. The serum was obtained by centrifuging the blood samples for 15 min at 4°C at 3000 × g, which was stored in a cryogenic refrigerator at −20°C for the subsequent determination of the relevant indexes. A fully automated biochemical analyzer (AU5800, American Beckman Coulter Co., Ltd., United States) was used to measure serum biochemical parameters, including total cholesterol (TC), triglyceride (TG), alanine aminotransferase (ALT), aspartate aminotransferase (AST), total protein (TP), globulin (GLB), albumin (ALB), high-density lipoprotein (HDL), low-density lipoprotein (LDL) and alkaline phosphatase (ALP).

Serum samples stored in −20°C refrigerator were taken to determine serum total antioxidant capacity (T-AOC) and malondialdehyde (MDA) content and immunoglobulin A (IgA), immunoglobulin G (IgG) and immunoglobulin M (IgM) according to the instructions of the kit produced by Nanjing Jianjian Bioengineering Institute (Nanjing, China).

### 2.5 Gut morphometric analyses

One hen of approximately average weight was randomly selected for slaughter in each replicate. The segments of the duodenum, jejunum, and ileum were taken, each approximately 2–3 cm in length, were rinsed with PBS solution and then fixed in a 10% paraformaldehyde solution. After 12 h, the solution was replaced and left to fix for over 24 h. The samples were embeded with the paraffin, then dewaxed after section, and stained with hematoxylin and eosin stains (H&E). The villus height (VH) and crypt depth (CD) were measured using a micrometer, and the villus height to crypt depth ratio (V/C) was calculated.

### 2.6 Statistical analyses

The results of indicators were all tested by t-test using the SPSS 22.0 software (SPSS Inc., Chicago, IL, United States). Statistically significant effects were further analyzed and means were compared by Duncan’s multiple range test. If *p* < 0.05, the difference was significant. All data in this study were presented as mean ± standard error of mean (SEM), and the graphs were generated by GraphPad Prism software 8.0 (GraphPad Inc., San Diego, United States).

## 3 Results

### 3.1 Performance and egg quality

Table 3 demonstrates that when compound probiotics were supplemented into the diet, LR and AEW were clearly raised (*p* < 0.05) and F/E was significantly lowered (*p* < 0.05) in comparison to CON. Nonetheless, there was no statistically significant difference (*p* > 0.05) in the ADFI of laying hens between PRO and CON.

**Table 3:** Effects of supplementation of compound probiotics on laying performance.

Items	CON	PRO	*p*-value
ADFI, g/d	119.80 ± 0.46	125.19 ± 3.81	0.260
LR, %	87.96 ± 2.05^b^	91.69 ± 1.70^a^	0.028
AEW / g	54.14 ± 1.09^b^	57.04 ± 1.24^a^	0.013
F/E	2.35 ± 0.07^a^	2.20 ± 0.05^b^	0.042

ADFI, average daily feed intake; LR, Laying rate; AEW, average egg weight; F/E, feed-to-egg ratio; CON, the control group; PRO, the treatment group. No letter or the same letter superscript indicates a nonsignificant difference (*p* > 0.05), whereas different small letters superscript indicates a significant difference (*p* < 0.05).

As shown in Table 4, with the supplementation with compound probiotics, YW, ESI, and AH of were significantly higher (*p* < 0.05) than CON, but the EP was significantly lower (*p* < 0.05). While between CON and PRO, there was no discernible difference in HU (*p* > 0.05).

### 3.2 Serum biochemistry

**Figure 1:**
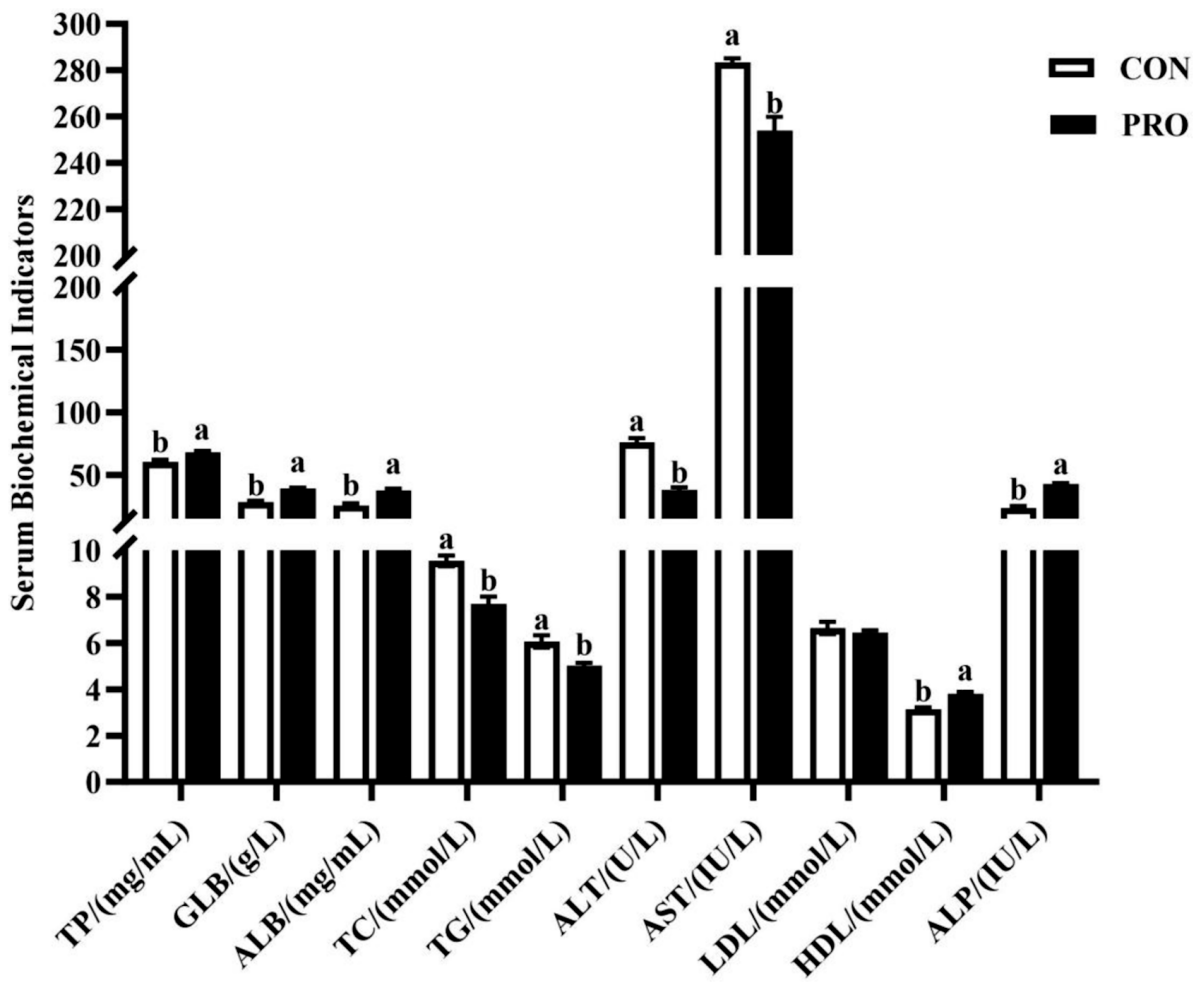
Effects of supplementation of compound probiotics on serum biochemical indicators of laying hens. TC, total cholesterol; TG, triglyceride; ALT, alanine aminotransferase; AST, aspartate aminotransferase; TP, total protein; GLB, globulin; ALB, albumin; HDL, high-density lipoprotein; LDL, low-density lipoprotein; ALP, alkaline phosphatase; CON, the control group; PRO, the treatment group. No letter or the same letter superscript indicates a nonsignificant difference (*p* > 0.05), whereas different small letters superscript indicates a significant difference (*p* < 0.05).

Figure 1 shows that the serum levels of TC, TG, ALT and AST significantly decreased (*p* < 0.05) in the in PRO. However, the serum levels of TP, GLB, ALB, HDL, and ALP significantly raised in PRO (*p* < 0.05).

**Table 4:** Effects of supplementation of compound probiotics on egg quality of laying hens.

Items	CON	PRO	*p*-value
YC	4.88 ± 0.13	5.63 ± 0.38	0.215
YW/g	15.06 ± 0.45^b^	16.60 ± 0.30^a^	0.013
ESI	1.27 ± 0.01^b^	1.33 ± 0.01^a^	0.037
ES/(kg/cm2)	51.90 ± 2.08	53.54 ± 1.92	0.331
YP/%	24.97 ± 0.27	25.72 ± 0.38	0.231
AP/%	63.24 ± 0.40	64.81 ± 0.47	0.070
EP/%	10.82 ± 0.05^a^	10.42 ± 0.05^b^	0.002
ET/mm	0.40 ± 0.01	0.42 ± 0.00	0.130
AH/mm	4.13 ± 0.59^b^	7.67 ± 0.26^a^	0.020
HU	77.32 ± 3.07	85.97 ± 0.69	0.085

YC, yolk color; YW, yolk weigh; ESI, egg shape index; ES, eggshell strength; YP, yolk proportion; AP, albumen proportion; EP, eggshell proportion; ET, eggshell thickness; AH, albumen height; HU, Haugh unit; CON, the control group; PRO, the treatment group. No letter or the same letter superscript indicates a nonsignificant difference (*p* > 0.05), whereas different small letters superscript indicates a significant difference (*p* < 0.05).

**Figure 2:**
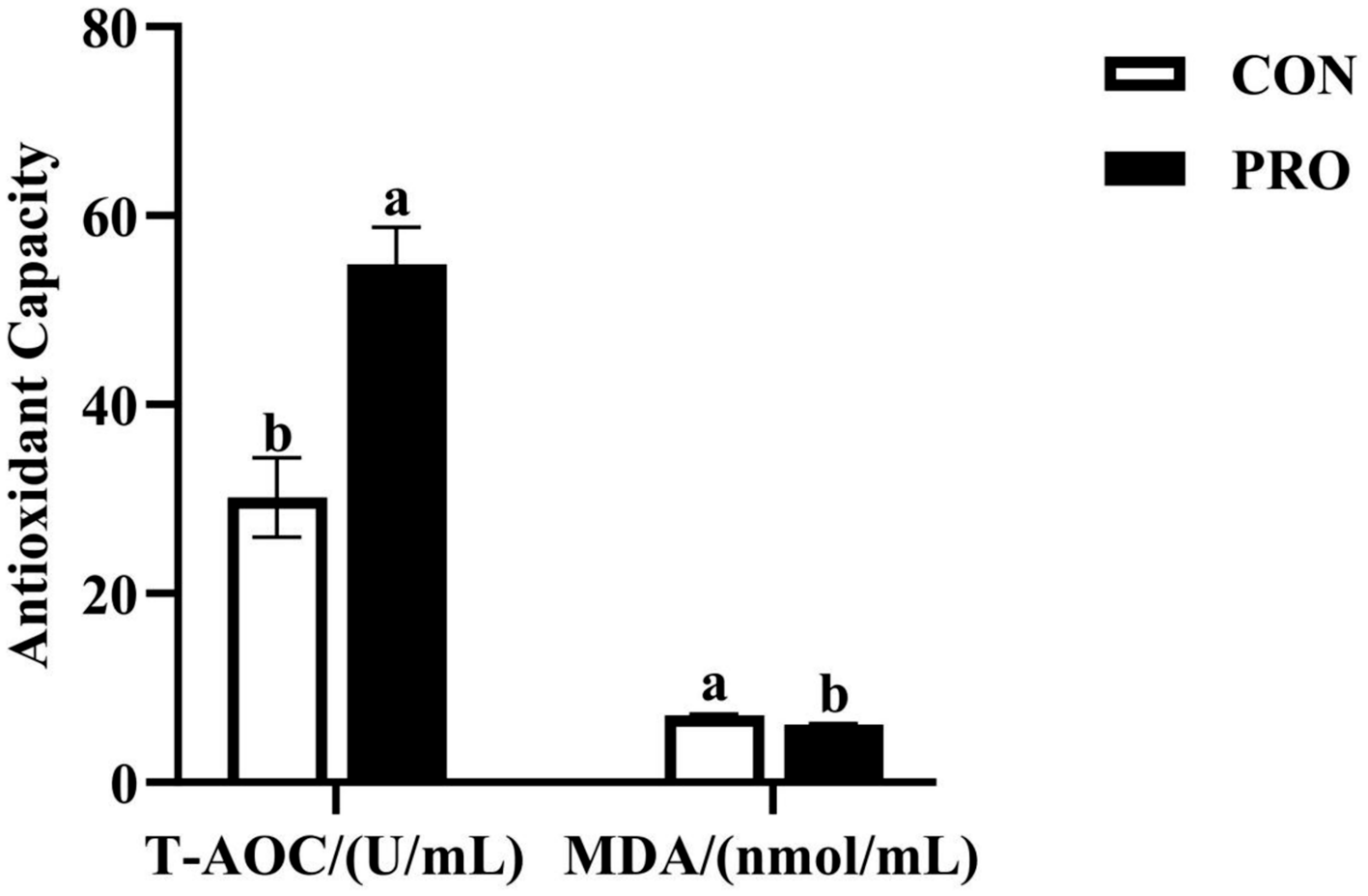
Effects of supplementation of compound probiotics on antioxidant capacity of laying hens. MDA, malondialdehyde; T-AOC, total antioxidant capacity; CON, the control group; PRO, the treatment group. No letter or the same letter superscript indicates a nonsignificant difference (*p* > 0.05), whereas different small letters superscript indicates a significant difference (*p* < 0.05).

**Figure 3:**
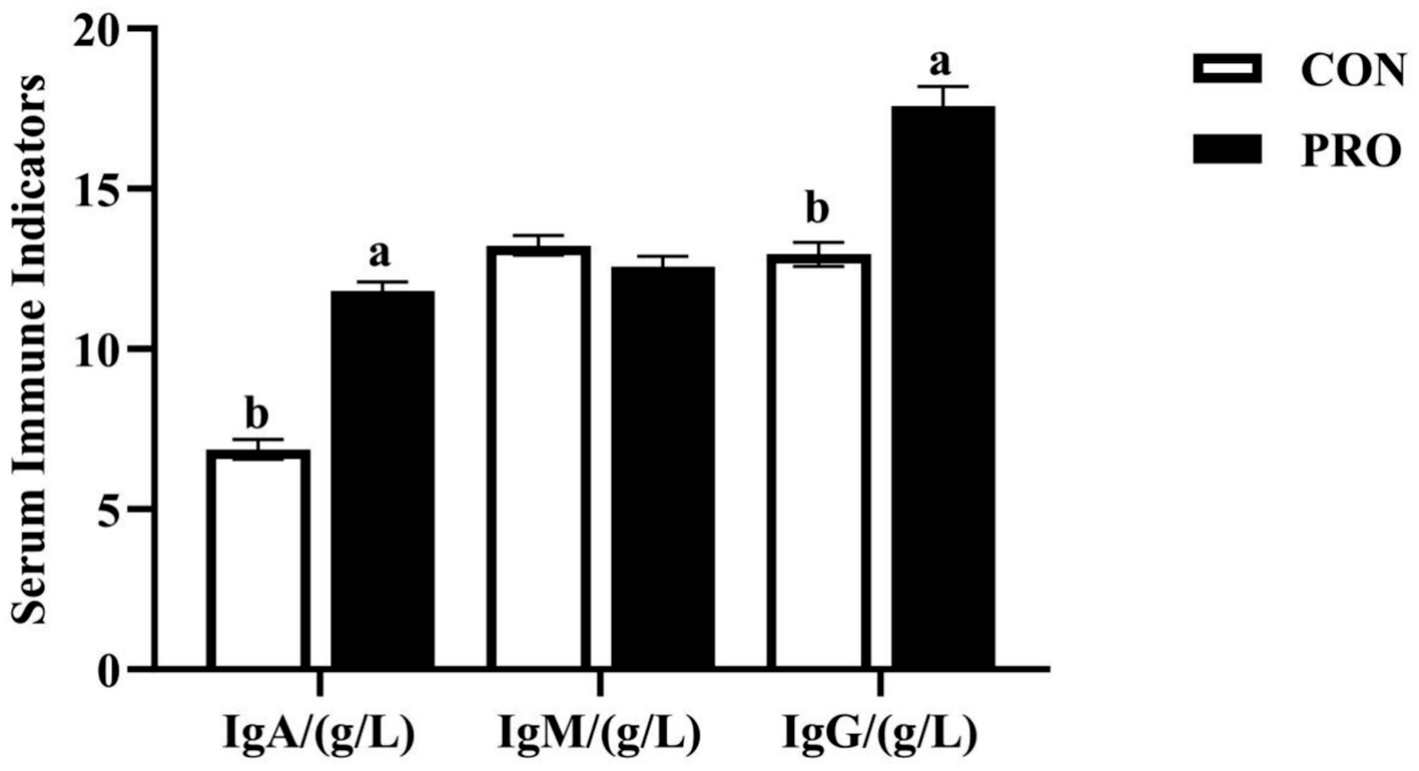
Effects of supplementation of compound probiotics on immune function of laying hens. IgA, immunoglobulin A; IgG, immunoglobulin G; IgM, immunoglobulin M; CON. the control group; PRO, the treatment group. No letter or the same letter superscript indicates a nonsignificant difference (*p* > 0.05), whereas different small letters superscript indicates a significant difference (*p* < 0.05).

As depicted in Figure 2, compared to CON, PRO had significantly less MDA (*p* < 0.05) and the T-AOC of PRO was significantly increased (*p* < 0.05). And Figure 3 demonstrates that while there was no significant difference in the IgM content between the CON and PRO (*p* > 0.05), the IgA and IgG levels in the serum of laying hens in PRO were considerably greater than those in CON (*p* < 0.05).

### 3.3 Gut morphometry analysis

**Figure 4:**
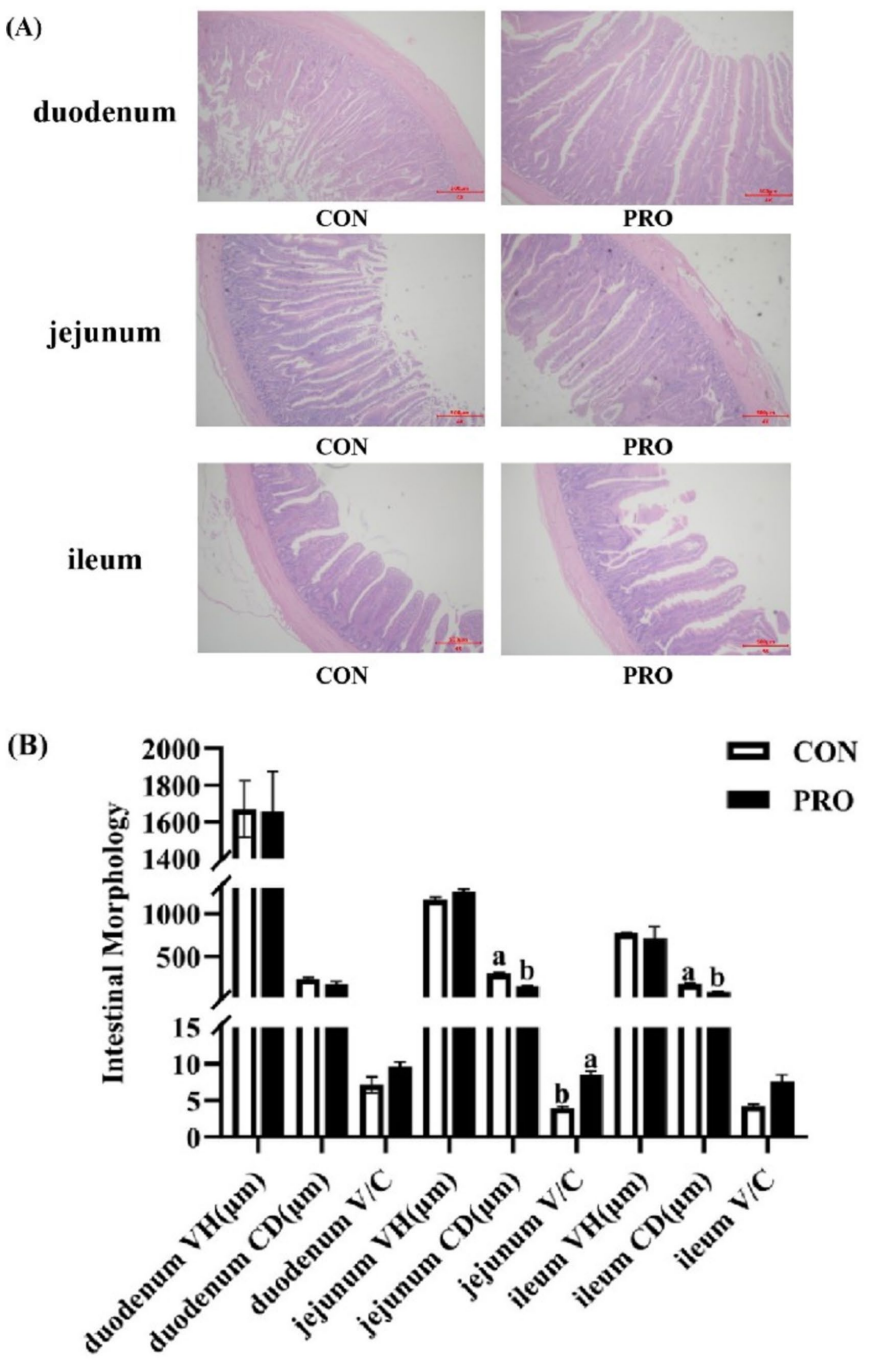
Effects of supplementation of compound probiotics on intestinal morphology of laying hens. **(A)** The morphology of duodenum, jejunum, ileum of the CON group and treatment group; **(B)** The VH, CD, V/C of duodenum, jejunum, ileum of the CON group and treatment group. VH, villus height; CD, crypt depth; V/C, villus height to crypt depth ratio; CON, the control group; PRO, the treatment group. No letter or the same letter superscript indicates a nonsignificant difference (*p* > 0.05), whereas different small letters superscript indicates a significant difference (*p* < 0.05).

Figure 4 presents that intestinal morphology of the duodenum in PRO did not differ substantially from that in CON (*p* > 0.05). However, compared to CON, the CD of the ileum and jejunum in PRO significantly decreased (*p* < 0.05) while the V/C of the jejunum in PRO significantly increased (*p* < 0.05).

## 4 Discussion

### 4.1 Performance and egg quality

In the context of intensive farming, the production performance and egg quality of laying hens are key indicators that affect the economic benefit of laying hens. And LR, AEW and F/E are key indicators for evaluating laying hens; production performance. As one of the most promising new feed additives after the ban on antibiotics, probiotics have a specific impact on the production performance of livestock and poultry after entering the intestine (25). Abdelqader et al. (26) reported that the dietary supplementation of *Bacillus subtilis* (2.3 × 10^8^ CFU/g) resulted in higher egg production and feed conversion ratio in hens. Previous studies showed that the addition of *Bacillus subtilis* culture (500 mg/kg) to laying hens can improve egg production and feed conversion rates (27). After the supplementation of a mixed culture of *L. acidophilus* and *L. casei*, *L. acidophilus*, or *B. subtilis* to the diet, egg production significantly increased (28, 29). The results of the present study were similar to those of the above studies in that the dietary supplementation of compound probiotics increased the LR and AEW while the F/E was reduced, which shows that compound probiotics as feed additives can improve the laying performance. This may be due to the fact that probiotics, after entering the intestinal tract, accelerate the utilization of feed nutrients by enhancing fiber digestion and enzyme activity. In contrast, some studies showed that the addition of 2.0 g/t of probiotics consisting of *Bacillus subtilis* and *Bacillus amyloliquefaciens* to the diet did not have a significant improvement on egg production and egg weight (30). Davis and Anderson (31) also found no significant increase in egg production after the supplementation of hens with products containing mixtures of bacteria such as *Lactobacillus* and *Bacillus*. These results differed from the results of the present study, which may be due to the fact that the effectiveness of probiotics application is limited by a number of factors such as microbial species composition, habitability, supplemental administration dose, method and frequency of application, dietary composition, age of the birds and environmental stress factors (32).

The quality of the eggshell is an important factor affecting the shelf-life of eggs, and about 10 to 15 per cent of eggs are damaged due to eggshell quality problems during egg collection, storage and transport. For example, the egg shape index directly determines the selection of breeding eggs and is positively correlated with hatching rate (33). And the eggs damaged due to weak eggshell quality account for 6–10% of all eggs produced worldwide, which causes huge economic losses (34). The study showed that the dietary supplementation with a mixture of *Lactobacillus* cultures resulted in a significant improvement in eggshell quality (35). However, in this study, the supplementation of compound probiotics did not significantly improve EW, ET and other indicators that can measure eggshell quality, which may be related to the amount of probiotics added, age and heritability of laying hens (36). Carvalho et al. and Ramasamy et al. showed that the supplementation of *Saccharomyces boulardii* (0.05 g/kg) and *Lactobacillus acidophilus* (0.1 g/kg) to the diet of laying hens did not improve eggshell quality (35, 36), this is in agreement with the results of the present study, indicating that the supplementation of compound probiotics had no adverse effect on the eggshell quality of the eggs. Moreover, in this study, the egg shape index of the eggs was also significantly improved by the supplementation of compound probiotics, which indicated that compound probiotics had an effect on calcium deposition in the eggshell, and also showed that the supplementation of compound probiotics could improve the eggshell quality of the eggs to a certain extent. The results of Cao et al. (37) showed a significant increase in egg shape index after the dietary supplementation of compound probiotics (1 g/kg), which is consistent with the results of the present study. Yolk weight and albumen weight were significantly and positively correlated with egg weight. Eggs with higher weights tended to have greater yolk quality compared to eggs with lower weights (38). In the conditions of this study, egg weight and yolk weight were significantly increased in the PRO group, indicating that the supplementation of compound probiotics can improve the nutritional value of eggs. AH is one of the important indicators used to evaluate the freshness of eggs (7). The result of Zhang et al. (39) showed that the addition of 5.6 mL of *Lactobacillus plantarum* (3 × 1,010 CFU/mL) per gram of basal diet significantly increased albumen height. Liu et al. (20) showed that the supplementation of yeast cultures (2 g/kg) to the diet significantly increased the AH of eggs. The results of the above study are in agreement with the results of the present study, where the dietary supplementation of compound probiotics significantly increased the albumen height, which may be attributed to the fact that the compound probiotics facilitated the synthesis and transport of proteins, which resulted in an enhancement in AH (40), and this result also demonstrates that the supplementation of compound probiotics improves the freshness of the eggs and thus effectively improves the egg quality.

### 4.2 Serum biochemistry

In serum, immunoglobulins play an important role in the immune function of the body, among which IgA, IgG and IgM are commonly used indicators to evaluate the immune status of the body (37). IgG has the role of neutralizing bacterial toxins and antimicrobial antitoxins, meanwhile, it has the highest content of all the immunoglobulins in the organism, and is an important antibody in the humoral immunity. IgM is capable of dissolving pathogenic bacteria. Probiotics have been shown to modulate serum systemic antibody responses to antigens and are involved in the development of immunity. Ahmed et al. demonstrated that the addition of *Bacillus amyloliquefaciens* to diets altered the immune response of broilers, with a linear improvement in serum heavy IgG and IgA levels (41). Previous studies on probiotics showed that 1.0 × 10^9^ CFU/day of *Bacillus subtilis* BS1 or 4.0 × 10^9^ CFU/ m^2^ of *Bacillus amyloliquefaciens* improves and diversifies the status of serum IgG, IgM, and IgA in broilers and mice and modulates the immune state (42, 43). The above studies were similar to the results of the present study, the supplementation of compound probiotics resulted in a significant increase of levels of IgA and IgG in serum, which improved the body’s immune defense mechanism to a certain extent. Probiotics have an important effect on the immune system of poultry against invading pathogens (44) and can stimulate innate and adaptive immunity by regulating the expression of toll-like receptors, activating dendritic cells and natural killer cells, increasing the response of t-helper cells, and inducing the production of cytokines and secretion of immunoglobulins, such as IgM, IgG and IgA (45).

Oxidative stress is an imbalance between oxidation and antioxidation in the body, which generates a variety of reactive oxygen species (ROS), damaging proteins, nucleic acids and lipids, leading to tissue damage and diseases (46). T-AOC is an important comprehensive indicator of antioxidant capacity (47). Antioxidant enzymes (SOD, CAT, GSH and GSH-Px) are the first line of ROS elimination, whereas MDA is the end product of lipid peroxidation and is often used as a biomarker to assess oxidative stress (48). There is a close relationship between the antioxidant capacity of the body defense system and the health degree. Superoxide dismutase can clear superoxide anion radical (O_2_^−^·) disproportionation to generate oxygen and hydrogen peroxide, then catalase can promote the decomposition of H_2_O_2_ into molecular oxygenated water to remove hydrogen peroxide in the body, thus protecting cells from damage (49). Qin et al. (50) showed that dietary supplementation with 300 mg/kg of probiotics did not increase the activity of antioxidant enzymes in the serum of laying hens, which is consistent with the results of the present study. However, the results of the present study showed that the MDA content decreased after the dietary supplementation of compound probiotics, which may be attributed to the ability of probiotics to secrete antioxidant peptides, which act as antioxidants with the ability to scavenge free radicals, such as ROS, and thus reduce the generation of lipid peroxides (51). The results of Xiang et al. showed that dietary supplementation of probiotics (1 g/kg) significantly reduced serum MDA levels, which is consistent with the results of the present study. Furthermore, the results of the present study also showed that the total antioxidant capacity (T-AOC) was significantly higher in the PRO group, the results of Fu et al. (52) similarly showed a significant increase in total antioxidant capacity with the dietary supplementation of probiotics. Thus, the improvement in serum T-AOC indicates that supplementation with compound probiotics improves antioxidant capacity and enhances innate immunity (53).

The content of TP, ALB and GLB in serum reflects the digestion, absorption and metabolism of feed proteins (54). The results of this study showed that the contents of TP, ALB and GLB were significantly increased after the supplementation of compound probiotics, indicating that the protein metabolism in the animal’s organism was vigorous, and it was able to better intake and utilize the feed proteins, which led to the reduction of the FER. Albumin is mainly synthesized by the liver, and has the role of protecting globulin and transporting metabolites in the organism, and the improvement in ALB content in this experiment may be attributed to the fact that the compound probiotics inhibited the growth of pathogens, which reduced the degradation of proteins, and improved the utilization rate of proteins in the feed (55). In addition, Abdel-Moneim et al. (56) showed that the supplementation of probiotics was able to enhance mucosal immunity and nutrient absorption by the animal organism, thereby, increased the level of immunoglobulin in the serum. Therefore, the results of this study indicate that the supplementation of compound probiotics of the basal diet can promote the ability of the organism to protein intake and utilization, improve the nutritional level of the organism, and thus enhance the immunity of the organism.

Probiotics have been shown to influence cholesterol metabolism by reducing cholesterol levels (57). According to our study, the serum cholesterol level of laying hens in the PRO group was significantly lower than that of the CON group. Loh et al. (58) reported that the dietary supplementation with 0.6% *Lactobacillus plantarum* reduced blood cholesterol levels in Pengging ducks. Qiu et al. (59) showed that dietary supplementation with low doses of probiotics reduced serum triglyceride concentrations in broilers. The above results are in agreement with the present study, which suggests that the dietary supplementation of compound probiotics can positively affect lipid metabolism and regulate triglyceride levels, thereby improving health status. Probiotics increase serum lipase activity, and in the presence of lipase, fat in the body is broken down by lipase into small molecules of fatty acids and glycerol, and excess fat is oxidatively broken down and converted into adenine nucleosides for direct consumption (60). And this may also be one of the reasons that the supplementation of compound probiotics can improve the lipid metabolism in laying hens. HDL regulates cholesterol levels and prevents cholesterol from accumulating in cells, and sterols are shed from membranes at the same rate as cholesterol synthesized by the liver to maintain a balance (61). The function of HDL is to transport unused surplus cholesterol to the liver. The remaining cholesterol will be used as a component in the production of steroid hormones and bile salts, while the remaining inactive cholesterol will be excreted from the body (62). In the study, the serum level of HDL was higher in laying hens in the PRO group. Mohamed et al. showed a significant enhancement in serum HDL levels with the supplementation of probiotics (500 mg/kg) to the diet, which is in agreement with the result of the present study (63). This indicates that the dietary supplementation of compound probiotics can regulate the level of lipid metabolism to a certain degree, which improves the health of the organism and enhances the laying hens’ performance of laying hens. ALT and AST levels are commonly used to reflect the health of the liver in animals, and elevated levels of both can indicate different degrees of liver damage (64). The results of the present study showed a significant reduction in the levels of AST and ALT by the dietary supplementation with compound probiotics. The study conducted by Chen (65) showed that feeding hens a diet containing *Lactobacillus* cultures greatly reduced the levels of ALT and AST. Liu et al. (20) showed that the addition of *yeast* cultures showed a significant reduction in AST levels, which is a positive sign of liver function. The results of our experiment are basically consistent with the above studies, suggesting that the supplementation of compound probiotics in the diet enhanced the repair and regeneration ability of the liver, thus playing a protective role for the liver.

### 4.3 Gut morphometry analysis

The intestinal tract is the main region of the organism for nutrient absorption, and the integrity of its barrier structure and function is a prerequisite for the diet to be fully digested and absorbed. Additionally, the primary indices for assessing the intestinal barrier’s structural and functional integrity are VH, CD, and V/C, which represent the intestinal tract’s capacity for absorption and digestion (66). VH and cell number are positively correlated, and the increase of VH expands the absorptive area of the small intestine, so the more mature epithelial cells and the shallower the CD, the better the intestinal digestion and absorption of nutrients (67). The V/C ratio is a measure of the digestive and absorptive capacity of the small intestine, and an improvement in the V/C ratio indicates an improvement in the intestinal mucosa and a greater intestinal capacity for digestion and absorption of nutrients (68). The supplementation of probiotics to the diet of laying hens significantly increased jejunal and ileal VH (69). The supplementation of *yeast* polysaccharides to the diet significantly increased VH of jejunum and ileum (70). The results of this experiment showed that the supplementation of compound probiotics to the diet of laying hens significantly decreased the CD of jejunum and ileum, while the V/C of jejunum was significantly increased, which may be attributed to the fact that the supplementation of compound probiotics could provide the nutrient requirements for the cell division and proliferation of intestinal tissues of the livestock and poultry, promote the development of the intestinal epithelial cells, and enhance the absorption ability of intestinal mucosa. Simultaneously, the experiment’s findings point to a possible connection between improved intestinal morphology and nutrient absorption function and the ability of compound probiotics to enhance laying efficiency and egg quality.

## 5 Conclusion

The results of this study showed that the dietary supplementation of compound probiotics of laying hens was able to regulate lipid metabolism pathways, reduce the degree of lipid peroxidation in the body, and thus improve the total antioxidant capacity of the body. In addition, in this study, the compound probiotics was able to regulate protein synthesis and improve intestinal morphology, which improved the immune performance of laying hens, and ultimately had a positive impact on laying rate and egg quality of laying hens. At the same time, this study also found that the supplementation of compound probiotics could reduce the F/E and improve the economic benefit of laying hens. In conclusion, compound probiotics have great potential in the field of ‘anti-antibiotic’ feed additives for laying hens and are worthy of further research.

## Data Availability

The original contributions presented in the study are included in the article/supplementary material, further inquiries can be directed to the corresponding authors.
